# Understanding Messaging Preferences to Inform Development of Mobile Goal-Directed Behavioral Interventions

**DOI:** 10.2196/jmir.2945

**Published:** 2014-02-05

**Authors:** Frederick Muench, Katherine van Stolk-Cooke, Jon Morgenstern, Alexis N Kuerbis, Kendra Markle

**Affiliations:** ^1^CASPIRDepartment of PsychiatryColumbia University College of Physicians & SurgeonsNew York, NYUnited States; ^2^CASPIRMental Health Services & Policy ResearchResearch Foundation of Mental HygieneNew York City, NYUnited States; ^3^Stanford Prevention LabStanford Prevention Research CenterStanford University School of MedicineStanford, CAUnited States

**Keywords:** mHealth, text messaging, behavioral health, preferences, linguistics, tailoring, participatory design, agile design

## Abstract

**Background:**

Mobile messaging interventions have been shown to improve outcomes across a number of mental health and health-related conditions, but there are still significant gaps in our knowledge of how to construct and deliver the most effective brief messaging interventions. Little is known about the ways in which subtle linguistic variations in message content can affect user receptivity and preferences.

**Objective:**

The aim of this study was to determine whether any global messaging preferences existed for different types of language content, and how certain characteristics moderate those preferences, in an effort to inform the development of mobile messaging interventions.

**Methods:**

This study examined user preferences for messages within 22 content groupings. Groupings were presented online in dyads of short messages that were identical in their subject matter, but structurally or linguistically varied. Participants were 277 individuals residing in the United States who were recruited and compensated through Amazon’s Mechanical Turk (MTurk) system. Participants were instructed to select the message in each dyad that they would prefer to receive to help them achieve a personal goal of their choosing.

**Results:**

Results indicate global preferences of more than 75% of subjects for certain types of messages, such as those that were grammatically correct, free of *textese*, benefit-oriented, polite, nonaggressive, and directive as opposed to passive, among others. For several classes of messages, few or no clear global preferences were found. There were few personality- and trait-based moderators of message preferences, but subtle manipulations of message structure, such as changing “Try to…” to “You might want to try to…” affected message choice.

**Conclusions:**

The results indicate that individuals are sensitive to variations in the linguistic content of text messages designed to help them achieve a personal goal and, in some cases, have clear preferences for one type of message over another. Global preferences were indicated for messages that contained accurate spelling and grammar, as well as messages that emphasize the positive over the negative. Research implications and a guide for developing short messages for goal-directed behaviors are presented in this paper.

## Introduction

Over the past decade, mental health researchers have sought to harness popular contemporary technologies, such as computers and mobile phones, in order to develop effective interventions for a range of medical and behavioral problems. The widespread availability and real-time potential of mobile phone-based short message service (SMS) has made SMS interventions an attractive and promising subject of investigation within this area. Numerous studies have shown that SMS interventions can improve outcomes across a variety of physical and mental health disorders [1-3]. SMS interventions have yielded small to moderate effects against no treatment controls, with text messaging for smoking cessation and HIV medication adherence yielding the largest effects [1]. Recent reviews suggest that the inclusion of SMS and other prompts improves the effects of Web-based interventions and highlights the value of mobile messaging to enhance many types of interventions [4]. Moreover, brief text-based interventions of 140 characters generalize to interventions that integrate Twitter and smart phone applications, for which similar character limits apply.

The content of SMS interventions has typically been based upon prevailing global behavior change theories, such as the transtheoretical model of behavior change, social cognitive theory [5], or, more recently, specific theory-based mobile intervention mapping techniques [6]. While these behavior theories represent a foundation for present and future research on mobile interventions, many other important intervention design features have been neglected. Some digital intervention development studies have focused on human-computer interaction [7] and persuasive design features [8] that emphasize subtle differences in nonspecific intervention components like tone, design, and structure to increase user engagement in the intervention.

The tone and structure of a message can have an impact on user receptivity and engagement in an intervention, as each point of contact is an opportunity to engage the end user. A few pioneering studies have examined how message framing impacts intervention outcomes or adherence to interventions. For example, Bickmore and colleagues [9] found that using empathy in computer interactions led to a more positive user experience, while perceived politeness of task interruptions by a virtual agent predicted long-term adherence. Yet another study found that individuals exercised more when their virtual agent was serious rather than playful [10], indicating that the overall tone of an intervention can have an impact on discrete behavioral outcomes and receptivity.

There is extensive literature on the benefits of tailoring computer-based intervention content, preferences, and feedback to individual users for health outcomes across conditions [11]. Intervention tailoring increases receptivity, memory for messages, self-relevance, and self-referential processing of information for specific subgroups [12,13]. Some of the earliest work on tailoring revealed that the ideal message type (eg, picture vs text) varies based on an individual’s need for cognition [14,15], indicating that individuals process content differently and will be more receptive to some forms of message presentation than others. Despite the wealth of research on intervention tailoring, there has been almost no research to suggest which types of individuals prefer which types of message structure and content. For example, do older individuals have more difficulty processing *textese* than younger individuals? Are women more receptive to emoticons in health messages than men [16]? To date, no research has examined how demographic variables may differentially impact preferences for goal-directed short text-based interventions.

Within the general intervention development field, several development studies have used focus groups and post-pilot interviewing to examine preferences for certain types of messages. For example, participants in an SMS intervention to promote weight loss disliked the inclusion of *textese* (eg, How r u feeling 2day?) and passive language in health messaging [17]. Multiple studies across a variety of health topics have further indicated that users prefer messages that are positive in tone or benefit-oriented, brief, and direct [17,18]. Moving beyond user preferences, some recent research has specifically focused on the impact of the underlying characteristics of text on readability and retention [19,20]. For example, Leroy and colleagues [20] revealed that, although low noun-phrase complexity was perceived by users as the simplest, grammatical manipulations had little impact on the readability of the content. These studies collectively highlight the importance of understanding the impact these message features can have on user receptivity.

A useful, cost-effective method for collecting this information is rapid and iterative user preference or beta testing using quantitative methods to combat the limitations of qualitative testing. These methods have been used often in consumer research to compile data on user engagement [21] or for public health campaign engagement [22]. In our previous work developing an SMS intervention for addiction continuing care, we compared preferences for benefit-oriented vs consequence-oriented messaging and found that individuals generally preferred benefit-oriented messages, but that message preference was moderated by the perceived benefits of being drug-free [23]. This research reinforced the tailoring outcome research on the importance of congruence between motivational processes and message framing. However, along with the consumer preferences research, it also revealed that preference research may be a useful tool for initial intervention development work in resource-limited environments.

This study examined preferences for a range of text messages designed to foster goal-directed behaviors. Text messages were displayed in mirrored dyads to present participants with variations in syntax and language, tone, locus of authority, and grammatical person. The aim of this study was to determine whether any global messaging preferences existed and how certain characteristics moderate those preferences in an effort to inform the development of mobile messaging interventions. In addition, we employed iterative design techniques to assess how subtle changes in messages affected preferences from one sample to the next. This study was approved by the New York State Psychiatric Institute Institutional Review Board (NYPSI IRB) and was part of the pilot intervention development work for a mobile adaptive alcohol intervention.

## Methods

### Recruitment

Participants were recruited online through Amazon.com, Inc.’s online labor market, Amazon Mechanical Turk (MTurk). MTurk is a communication platform through which *workers* can be contracted to perform tasks that require human intelligence (eg, consumer surveys or beta testing) in exchange for compensation by the *requesters* who published the tasks. These tasks—called *human intelligence tasks* (HITs)—can range from one brief question to a 30-minute survey. Over the last few years, MTurk has been used for social sciences research with results similar to other sampling methods when certain validity checks were included in the design [24].

### Study Eligibility

MTurk worker qualifications for this study included a HIT approval rate of 95% or greater out of at least 500 completed HITs. This ensured a sample of workers whose work on previous HITs had been consistently deemed acceptable by other requesters, as well as a sample who demonstrated an appropriate degree of computer and Internet literacy. The subject pool was further limited to participants who were located in the United States. Workers who met these qualifications could view our HIT, titled *Answer a survey about your text message preferences,* and published through our requester account, *Columbia University Research*. Eligible workers could follow a Web link to an external, Web-based survey hosted by Survey Monkey, which has been used as a survey host in numerous research studies. Prior to completing the survey, participants completed a brief consent form for anonymous survey-based research, which also provided investigator and IRB contact information. In the consent form, participants were informed that the study’s aim was to understand the types of text messages they would prefer to receive when trying to achieve a personal goal. Once participants completed the survey, they were provided with a survey code to enter into their MTurk account to await requester review and compensation. Only participants referred through MTurk received compensation.

For the purposes of maintaining anonymity, we could not link the survey to the participants’ MTurk accounts, but included several a priori validity checks for anonymous survey research in both the survey and our MTurk requester account. These validity checks were included in accordance with the Checklist for Reporting Results of Internet E-Surveys (CHERRIES) [25]. We published the survey four times, counterbalancing the multiple choice options and reverse ordering questions. In Survey Monkey, our safeguards included blocking IP addresses once the survey was opened by a worker in order to bar them from retaking it, omitting responses of users who did not type cogent responses to open-ended questions, and/or gave conflicting answers to a duplicated message preference question. Although we were unable to match an individual worker to his or her survey responses, we were able to view the total amount of time each worker spent on the HIT in MTurk. As our survey should take a minimum of 6 minutes to complete, we rejected the work of participants who spent fewer than 6 minutes completing it.

### Participants

In total, 452 participants took one of four message preference surveys. Of those, 98 were not included in this paper because they were not located in the United States. These participants were primarily located in India and will be discussed in another paper. Of the 354 US participants, 277 were included in the final sample: 58 were excluded due to conflicting responses to identical but counterbalanced items, 9 due to missing or illegible goals, and 10 due to survey completion in under 6 minutes.

### Assessments

The assessment contained approximately 90 items, which were presented in groups of approximately 8 items per screen. Participants were asked to supply a personal goal they would like to achieve and to choose one of two messages in each dyad that they would prefer to receive to help them achieve that goal. These goals did not have to be health related. Participants were told that their goal could be anything from exercise to flossing more to being more assertive, and that there were no wrong goals. There were approximately 70 message dyads in 22 groupings. Each grouping typically consisted of three dyads. Message dyads were based primarily on pre-existing motivational and behavior change content and linguistic differences in message presentation derived from previous messaging studies, public health messaging campaigns, and our own experience writing messages. Based on these existing messages, we developed a corresponding, mirrored message to test a specific preference. For example, if a message included the word *you*, we then created a mirrored message with the word “we” in place of “you”. Organizational constructs for message design, message groupings and descriptions, an example dyad for each grouping, and the number of dyads per grouping are presented in Table 1. In addition, we included several single item semantic differential dyads on personality or disposition. These included face valid dichotomous semantic differentials (eg, I tend to be a sad person/I tend to be a happy person; I get along well with others/I have trouble getting along with others; I often get frustrated with the behavior of others/I don’t let what others do bother me much).

**Table 1:** Message groupings.

Organizing Principle	Dyad Grouping	Grouping Description	Dyad Example	Dyads/ Grouping
**Gain Framing vs Loss Framing**				
	**Smiley Emoticon vs Sad Emoticon**			
		Smiley Emoticon messages contain a smiley face to make the content gain-framed.	Don’t give up :-)	3
		Sad Emoticon messages contain a sad-face to make the content loss-framed.	Don’t give up :-(	
	**Benefit-Oriented vs Consequence-Oriented**			
		Benefit-Oriented messages consist of language that is gain-framed.	Close your eyes – imagine the benefits of changing.	3
		Consequence-Oriented messages consist of language that is loss-framed.	Close your eyes – imagine the consequences if you don’t change.	
**Personal/Emotional Emphasis**				
	**Coaching vs Uncoached Direction**			
		Coaching messages contain a direction or recommendation with positively framed emotional emphasis.	You’ve been doing great, don’t quit now.	3
		Uncoached Direction messages contain a direction or recommendation with no additional emphasis.	The most important thing you can do to reach your goal is not give up.	
**Goal-Setting and Task Performance**				
	**Implementation Intention vs General Goal**			
		Implementation Intention messages consist of an if-then plan to trigger a specific action.	If I start to get down on myself, I will think of all my previous successes.	3
		General Goal messages consist of an open-ended, nonspecific if-then plan.	If I start to get down on myself, I will do something to make me feel better.	
**Locus of Control Theory**				
	**Intrinsic Locus of Control vs Extrinsic Locus of Control**			
		Intrinsic Locus of Control messages emphasize an internal locus of control over goal attainment.	You are responsible when you don’t meet your goal.	4
		Extrinsic Locus of Control messages emphasize the degree to which external factors influence goal attainment.	Many different aspects of your environment play a role when you don’t meet your goal.	
**Spelling and Grammatical Manipulations**				
	**Correct Grammar vs Grammatical Errors**			
		Correct Grammar messages contain no grammatical errors.	If you accept where you are now, you’re way ahead of the pack.	3
		Grammatical Error messages contain grammatical errors.	If you accept where you are now you’re way ahead of the pack.	
	***Textese* vs *Non-Textese***			
		*Textese* messages utilize the spelling abbreviations common to text messaging.	u have changed b4, u can meet ur goals today. b who u r.	3
		Non-*Textese* messages	You have changed before, you can meet your goals today. Be who you are.	
**Manipulations of Visible Emphasis**				
	**Single Punctuation vs Multiple Punctuation**			
		Single Punctuation messages utilize only a single punctuation mark between phrases or clauses.	Reinvent yourself!	4
		Multiple Punctuation messages utilize multiple punctuation marks between phrases or clauses for emphasis.	Reinvent yourself!!!	
	**Smiley Emoticon vs No Emoticon**			
		Smiley Emoticon messages contain a smiley face to enhance a friendly or positive tone.	You are on the right track :-) just keep going!	3
		No Emoticon messages contain the same language as their Smiley Emoticon counterparts, but do not include an emoticon.	You are on the right track – just keep going!	
	**CAPS (capitalization) Emphasis vs No Visible Emphasis**			
		CAPS Emphasis messages contain at least one world that is spelled in all capital letters for emphasis.	When it comes to the negative consequences of a bad habit, you are NOT the exception.	4
		No Visible Emphasis messages do not include any all-caps words.	When it comes to the negative consequences of a bad habit, you are not the exception.	
**Manipulations of Voice, Person or Origin**				
	“**I” Statement vs “We” Statement**			
		“I” Statement messages employ a singular first person point of view.	Changing can be hard: I promise it will get better.	4
		“We” Statement messages employ a plural first person (or collectivist) point of view.	Changing can be hard: we promise it will get better.	
	“**You” Statement vs “We” Statement**			
		“You” Statement messages employ a singular second person point of view.	Your past should motivate you to change – not paralyze you!	
		“We” Statement messages employ a plural first person (or collectivist) point of view.	Our pasts should motivate us to change – not paralyze us!	
	**Male Quote vs Female Quote**			
		Male Quote messages consist of a quote from a famous man.	“When it is darkest, men see the stars.” Ralph Waldo Emerson	2
		Female Quote messages consist of a quote from a famous woman.	“I like the night. Without the dark, we’d never see the stars.” Stephanie Meyer	
	**Cited vs Uncited**			
		Cited messages refer to a source/sources of the information presented.	Studies show that simply visualizing your future actions makes them more likely to come true!	3
		Uncited messages provide no point of reference for the information presented.	Simply visualizing your future actions makes them more likely to come true!	
**Manipulations of Tone**				
	**Direction vs Passive**			
		Direction messages express a command.	Think about what you will lose if you give up on your goals.	3
		Passive messages express a suggestion in a passive or non-urgent tone.	It could be helpful to think about what you will lose if you give up on your goals.	
	**Statement vs Question**			
		Statement messages utilize declarative language.	Committing to your goals today will help you in the long-run.	4
		Question messages utilize interrogative language.	How will committing to your goals today help you in the long-run?	
	**Aggression vs Nonaggression**			
		Aggression messages utilize a confrontational or shaming tone.	Do you seriously think that blaming others will help you change for the better?	3
		Nonaggression messages utilize a non-confrontational tone.	Blaming others probably won’t help you change for the better.	
	**Polite vs Non-Polite**			
		Polite messages include words such as *please* and *thank you*.	Please text us to let us know if you received this message.	2
		Non-Polite messages do not include words such as *please* and *thank you.*	Text us to let us know if you received this message.	
	**Directive vs Nondirective Statement**			
		Directive messages contain an imperative statement within the context of a time frame.	Call a friend to help you feel better as soon as you have a free moment.	3
		Nondirective Statement messages offer suggestions with no direction or time-sensitive context.	Going out with friends is a good idea to help you feel better.	
	**Humor vs Gravity**			
		Humor messages include a joke or playful tone to suggest levity.	Why did the chicken cross the road? Because it knew that action creates change.	2
		Gravity messages are serious in tone and do not contain playful or jocular language.	Action creates change.	
**Symbolic Language**				
	**Metaphor vs Literal**			
		Metaphor messages contain symbolic imagery.	When you reach the end of your rope, tie a knot and hang on.	5
		Literal messages present content in plain terms.	When you feel like giving up, keep going until it passes.	
**Brevity vs Added Meaning**				
	**Short vs Long**			
		Short messages contain as little content as possible to convey meaning.	Your actions define you.	3
		Long messages are designed to convey additional meaning.	Your actions define you: the world looks at you differently when you act differently.	

The survey was published on MTurk a total of 4 times. After the data from the first 2 survey publications was downloaded and analyzed, a number of message dyads and groupings were removed if there appeared to be a clear consensus in preference among participants (eg, the Smiley Emoticon vs Sad Emoticon grouping). New dyads and categories were then added to the survey for publication on MTurk the third and fourth time. These revisions account for the differences in the sample size for many of the message groupings examined. The content of 3 message dyads within 2 groupings was slightly altered over the course of the study in order to correct for vagueness, disproportionately weighted language, or language that did not accurately reflect the general profile of a message grouping. Specifically, dyad #3 in the Directive vs Passive grouping and dyads #2 and #3 in the Statistic vs Anecdote grouping were altered. Ultimately, the Statistic vs Anecdote grouping was excluded from the main findings due to the researchers’ concern that the grouping as a whole was unsound. Therefore, only differences based on the alterations made in dyads #2 and 3 in this grouping are reported. Goals were coded into three broad categories based on their subject matter: physical health and well-being, competence and mastery, and personal fulfillment. Goals within these categories were then subcoded into more specific groupings as follows. In the physical health and well-being category, goals were subcoded as weight loss, fitness, nutrition, smoking cessation, sleep health, or personal hygiene goals. In the competence and mastery category, goals were subcoded as professional, academic, financial, or personal goals. In the personal fulfillment category, goals were subcoded as emotional, social, or spiritual goals. Finally, we included process rulers related to one’s self-selected goal such as goal importance, benefits of meeting that goal, and goal efficacy, which have been used in previous research [26].

### Data Analysis

A dichotomous variable for preferences within each dyad grouping was created based on a participant’s majority preference for messages in that dyad (ie, at least 2/3 or 3/4 messages chosen). If a category included four messages, individuals who chose two messages of each type (50/50 preference) were removed from analysis. Moderator analysis was conducted using chi-square analysis and comparative percentages are reported.

## Results

### Overview

Demographics are presented in Table 2. Overall, the sample was predominately white and middle aged with at least a high school degree, and 130 out of 277 participants (47.1%) were working full-time. Most had phone plans with SMS capabilities, and 197 out of 277 (71.1%) had unlimited texting plans.

**Table 2:** Demographics (n=277).

Variable		n (%)
**Age (years)**		
	18-30	113 (40.8)
	31-40	90 (32.5)
	41-older	74 (26.7)
Gender (% female)		156 (56.5)
**Race**		
	Black	19 (6.8)
	White	225 (81.1)
	Asian	20 (7.1)
	Other	12 (5.0)
**Ethnicity**		
	Hispanic	22 (8.2)
**Education level**		
	High School or GED	33 (12.1)
	Some College	77 (28.2)
	College Degree	123 (45.1)
	Graduate Degree	40 (14.7)
Employment status (% employed full-time)		130 (47.3)
Phone plan includes text messaging		266 (96.1)
Type of text messaging plan (% unlimited)		197 (71.2)

### Personal Goals

Participants generated a variety of personal goals to refer to while choosing their preferred messages. In total, 137 out of 277 participants (49.5%) generated personal goals related to physical health and well-being. Within this broad category, 52 out of 137 participants (38.0%) generated fitness goals (eg, “I want to go to the gym more often”) and 44 out of 137 participants (32.1%) generated weight loss goals. Also, 103 out of 277 (37.2%) participants generated personal goals related to competence and mastery. Within this category, 33 of 103 participants (29.9%) generated financial goals (eg, “I want to save more money this year”), 25 of 103 participants (24.3%) generated professional goals (eg, “I want to advance in my company”), and 18 of 103 participants (17.4%) generated personal mastery goals (eg, “I want to build my own house”). In addition, 33 out of 277 participants (11.9%) generated goals related to personal fulfillment (eg, “I want to communicate more effectively with my spouse” or “I want to have more fun”).

### Message Preferences

Results of messaging preferences are presented in Table 3. For the intents and purposes of this paper, we define “clear preference” as a preference of 75% or more of respondents for one message type within a grouping. There were clear preferences for about half of the groupings, with more than 90% of respondents selecting messages that did not include textese, a sad emoticon, incorrect grammar, or an external locus of control in those four groupings. There were also strong global preferences for messages with a benefit-oriented or active tone and for polite messages. For many of the groupings, no clear preferences were found for the entire sample.

**Table 3:** Message grouping preferences^a^ (n=277).

Message Type: Greater Preference	%	Message Type: Lesser Preference	%	n^c^
Smiley emoticon	97.6	Sad emoticon	2.4	213^b^
Correct grammar	96.7	Grammatical errors	3.3	211^b^
Non-textese	95.8	Textese	4.2	216^b^
Locus of control: intrinsic	93.5	Locus of control: extrinsic	6.5	46^b^
Benefit-oriented	89.2	Consequence-oriented	10.8	195^b^
Polite	86.5	Impolite	13.5	208^b^
Nonaggression	82.9	Aggression	17.1	269^b^
Direction	82.3	Passive	17.7	211^b^
Statement	82.0	Question	18.0	245^b^
No Humor	77.9	Humor	22.1	188^b^
Male quote	71.9	Female quote	28.1	146^b^
“I” statement	66.7	“We” statement	33.3	264^b^
Single punctuation	64.9	Multiple punctuation	35.1	174^b^
“You” statement	62.9	“We” statement	37.1	272^b^
Uncited	62.9	Cited	37.1	272^b^
Nondirective	61.0	Command	39.0	272^b^
Coaching	57.4	Uncoached direction	42.6	61
Literal	56.6	Metaphorical	43.4	272^b^
Smiley emoticon	53.6	No emoticon	46.4	274
CAPS (capitalization) emphasis	53.1	No visible emphasis	46.9	213
General goal	52.6	Implementation intention	47.4	57
Short	51.1	Long	48.9	272

^a^See Table 1 for a definition and example of each dyad.

^b^Indicates that a message preference is not the result of chance using a non-parametric binomial test to ensure that the difference between groups was greater than a 50% chance (*P*<.05).

^c^The n applies to both message types.

### Message Revision

Because this was an intervention development study, we also created several messages in which we manipulated specific language components from sample to sample. Slightly altering the wording of the passive message within Directive vs Passive dyad #3 from “Every time you feel down, try to change your thoughts to something positive about change” to “Every time you feel down, you might want to try to change your thoughts to something positive about change” resulted in an increase in participants’ overall preferences for the directive message: 127 out of 208 participants (61.1%) preferred the directive message prior to the dyad’s change, but 51 out of 57 participants (89.5%) preferred the directive message post-change. Within a message grouping that examined preferences for Statistics vs Anecdotes, changing the statistic within message dyad #3 from “93% of people who monitor their food intake reduce their calorie intake” to “44% of people who monitor their food intake reduce their calorie intake” caused overall preferences for the statistic to diminish; while 156 out of 212 participants (73.6%) preferred the message containing a statistic in the first version, only 37 out of 60 participants (61.7%) preferred it after the statistic was changed. Conversely, changing the statistics in message dyad #4 from “People who report doing nice things for other people are 44% happier than those who do not” to “People who report doing nice things for other people are 93% happier than those who do not” caused the overall preferences for the statistic to increase: 97 out of 210 participants (46.2%) preferred the message containing a statistic prior to its change, while 37 out of 57 participants (64.9%) preferred the message after the statistic was changed.

### Message Preference Moderators

We assessed differences in preferences based on several demographic variables, including gender, age, and education. A significant difference existed between male and female participants’ preferences for messages in only one message grouping, with female participants being more likely than male participants to prefer correct grammar to incorrect grammar (χ^2^_212_=5.334, *P*=.021; male=93.5%, 86/92; female=99.2%, 120/121). Similarly, the only significant difference that existed between older and younger individuals’ preferences was that older individuals were more likely than younger individuals to prefer “you” statements to “we” statements (χ^2^_271_=7.669, *P*=.006; over 40 years=76.4%, 55/72; 40 years or under=58.0%, 116/200).

Significant differences in preference existed between participants with different levels of education for several message groupings as well. Participants with less than a college degree were more likely than participants with a college degree or greater level of education to prefer directions to suggestions (χ^2^_210_=6.061, *P*=.014; no college=97.9%, 95/97; college=89.5%, 102/114), short messages to long messages (χ^2^_267_=3.759, *P*=.053; no college=55.1%, 70/127; college=43.3%, 61/141), and messages that included smiley emoticons to messages that contained no emoticons (χ^2^_269_=3.569, *P*=.059; no college=59.4%, 76/128; college=47.9%, 68/142). We ran a multiple logistic regression with significant moderators as the independent variables and education as the dependent variable. When controlling for all variables, education only moderated the preferences for the direction vs passive suggestion grouping (Wald statistic _3,205_ =5.26, *P*=.022). There were no differences in message preferences based on employment status.

We also assessed differences in preference based upon personality or trait variables. Participants who reported being generally sad were significantly more likely than participants who reported being generally happy to prefer commands to nondirective general statements (χ^2^_267_=4.037, *P*=.045; sad=50.8%, 30/59; happy=36.4%, 76/209), literal language to metaphors (χ^2^_267_=6.508, *P*=.011; sad=70.7%, 40/58; happy=51.9%, 109/210), non-polite messages to polite messages (χ^2^_204_=3.907, *P*=.048; sad=22.7%, 10/44; happy=11.2%, 18/161), and loss-framed to gain-framed messages (χ^2^_190_=4.193, *P*=.041; sad=20.5%, 9/40; happy=8.6%, 13/151). There was also a trend for this group to prefer “I” statements to “We” statements (χ^2^_259_=3.136, *P*=.077; sad=76.8%, 43/56; happy=64.2%, 131/204). Multiple logistic regression with significant moderators revealed that only the preferences of “I” statements to “We” statements (Wald statistic _4,179_ =4.74, *P*=.029) and commands to nondirective general statements (Wald statistic _4,179_ =8.29, *P*=.004) remained significant.

Despite the fact that participants reported radically different goals, the only differences that existed between participants with different higher order goals were that those with personal fulfillment goals were significantly more likely to prefer consequence messages than either those with physical health and well-being or competence and mastery goals (χ^2^_189_ =6.829, *P*=.033; personal fulfillment=24.0%, 6/25; physical health and well-being=10.8%; 10/93; competence and mastery=5.5%, 4/73). However, a heavy preference for benefit-based messaging existed across all three groups. The relationship between goals and messaging preferences will be discussed at length in a future paper.

Finally, we assessed differences in preferences based on three process rulers: the participants’ perceived benefits of changing, confidence about their ability to change, and perceived importance of changing. A preference for coaching messages was significantly associated with perceiving greater benefits of change (F_1,31_=4.33, *P*=.047). A preference for caps (capitalization) emphasis was significantly associated with perceiving greater benefits of change (F_1,110_=4.719, *P*=.032), higher confidence in one’s ability to change (F_1,197_=6.732, *P*=.012), and perceiving greater importance of changing (F_1,200_=9.325, *P*=.003). When entered into a logistic regression and with the benefits of changing variable removed due to a small sample size, only goal importance remained significant (Wald statistic _2,197_ =6.78, *P*=.009) while goal confidence trended towards significance (Wald statistic _2,197_ =3.43, *P*=.064).

## Discussion

### Principal Findings

To our knowledge, this is the first study to quantitatively examine messaging preferences for a range of message types to help guide text-based mobile intervention development. Results of this study indicate that there are clear user preferences for certain types of message characteristics over others, underscoring the importance of attention to message structure, linguistic content, and overall tone in the development of messages for goal-directed behaviors. This is particularly true of accurate spelling and grammar, as well as messages that emphasize the positive over the negative. While there has been little quantitative research on this topic, the findings of the present study are generally reflective of past qualitative research on messaging development, and are further supported by the Centers for Disease Control and Prevention’s *Guide to Writing for Social Media* [27]. For guidelines on writing messages for goal-directed behaviors based on this research and other sources, please see Multimedia Appendix 1.

### Spelling, Punctuation, and Grammar

Participants indicated an overwhelming preference for messages that were accurately spelled and grammatically correct over messages that included *textese* or contained grammatical errors. *Textese* can be more difficult to process than properly spelled words and phrases [28]. It is possible that participants’ overwhelming preference for proper spelling over *textese* is due to the fact that *textese* can impede comprehension and therefore reduce message receptivity. Another explanation for this finding that has been suggested in qualitative studies is that the inclusion of *textese* or the accidental inclusion of spelling and grammatical errors threatens the source credibility of messages that are designed to help users achieve a goal [8,29]. This may also explain participants’ preferences for serious messages over messages that attempted to be humorous.

The third and fourth message groupings to examine message syntax compared messages with single punctuation (eg, “.” or “!”) to messages with multiple punctuation marks (eg, “…” or “!!!”) and capitalization of a whole word or phrase to no capitalization (eg, “When it comes to the negative consequences of a bad habit, you are NOT the exception”). Multiple punctuation marks or capitalization can be utilized to add emphasis or to create a pause, and thus constitute visual substitutions for verbal cues. Participants’ preferences for single over multiple punctuation marks was much less pronounced than their preferences for proper spelling and grammar, and there was no clear preference between messages with caps emphasis versus messages with no visible emphasis. However, participants who reported that meeting their goal was very important and would benefit them immensely were more likely to prefer messages with some or all capitalized words for emphasis, and there was a trend toward a significant preference for multiple punctuation marks in the high benefits of change group. This emphasizes that understanding the end user’s state is a crucial component of intervention development.

### Emoticons

We examined variations in preferences for two different message groupings that contained emoticons: one that compared messages with a smiley face to identical messages with a sad face, and another that compared messages with a smiley to messages with no emoticons. Of all of the message groupings we examined, preferences were strongest in the smiley versus sad emoticon message grouping, with participants vastly preferring messages with the smiley. This finding resonates with past research that suggests that users vastly prefer positive messages to negative messages, as does our finding that participants generally preferred benefit-oriented to consequence-oriented messages. By contrast, no clear preference existed for messages that contained a smiley versus messages that contained no emoticon. It is possible that some participants found the inclusion of an emoticon too informal within messages designed to help users achieve a personal goal, while others perceived the inclusion of a smiley face as encouraging or rewarding when compared to a message with no emoticon. While virtually all participants seem to prefer a positive to a negative image, the fact that preferences within the two emoticon groupings varied so extremely suggests that the inclusion of an emoticon can communicate very different things depending on the context.

In concordance with previous literature suggesting that visual cues are more effective than text for those with lower need for cognition [15,30], we found that individuals with less education were more likely to prefer the inclusion of an emoticon than those with more education. Understanding who may be more receptive to emoticons [31] is an important line of research due to the frequency in which they are integrated into existing health messaging interventions. When taken together, these results underscore the need to tailor communication patterns to individual differences to obtain maximum engagement in goal-directed interventions.

### Sentence Type: Declarative, Interrogative, and Imperative

The clear user preferences for statements over questions have particularly interesting implications, as self-evaluative questions are often used in order to integrate motivational interviewing techniques into messaging interventions. Based on our findings, the inclusion of such messages without a fuller understanding of the preferences of the end-user requires some reconsideration. For example, will an individual who drinks too much be motivated to contemplate and evaluate the consequences of drinking simply because a text message asks him or her to do so? Further, questions that do not require interactivity may be disregarded by the individual because they will receive no feedback on their response. On the other hand, Muench and colleagues [23] found that individuals who are in the process of thinking about engaging in problem behaviors requested self-evaluative messages, suggesting that participants may be more receptive to such questions at specific times or stages of change.

There were clear preferences for more directive language over passive or suggestive language. Moderator analysis revealed that the preference for directive language was especially pronounced in older adults and individuals with more education. This could be a result of several factors, but may simply indicate that these users have been taught to avoid passive or suggestive language when communicating. While participants’ overall preference for directive over passive messages was clear, they were more averse to commands for immediate action (eg, “Do x right now…”), indicating that while individuals may want instruction, they may not want to feel commanded to behave a certain way in the moment.

### Sentence Content, Grammatical Person, and Length

As mentioned above, there was also a general preference for messages that did not include humor. It could be that humor minimizes one’s struggle to achieve a goal and should be used sparingly and possibly only after an alliance is built. Messages that were presented in first person singular (“I” statements) and second person (“You” statements) were preferred to messages that were presented in first person plural (“We” statements). This may indicate that participants generally prefer to be identified as individuals as opposed to one of a number of people, and prefer to identify the message originator as an individual as well.

We found no clear preferences for several other types of messages within groupings, including short vs long messages. This grouping is of particular interest because intervention developers have often been encouraged to break down messages to their smallest component pieces [32]. By contrast, our findings suggest that there are instances in which shortening a message can constitute a sacrifice in its readability, meaning, or cohesiveness, which should be avoided where possible. While there was a general preference for uncited statements over cited statements, we found that increasing the percentage of the effect in a statistic (eg, 44% to 93%) resulted in more pronounced preferences for the statistic. This finding corresponds well to the research on conformity and the power of social norms to increase the persuasiveness of messages. It is also possible that there are significant differences in how different outcomes (eg, happiness vs mortality) might significantly alter the persuasiveness of differing statistics, and this should be explored in greater detail.

### Group Differences

Differential preference analysis was designed to help distinguish preferences among different groups. In our case, overall analyses did not reveal dramatic shifts in preferences, but rather subtle differences between certain groups on certain variables. For example, while younger participants were significantly more likely than older participants to prefer “We” messages to “You” messages, the two groups differed by only 14%. In fact, few moderators shifted one group’s overall preferences for a dyad from one message type to the other. However, the differences reported are significant and future research should examine these subtle variations in message preferences based on these differences. For example, Muench and colleagues [23] found that in a substance abuse sample, individuals generally preferred benefit-oriented messaging, but that this was more pronounced with individuals who reported higher perceived benefits of changing. Because this finding is similar to the general health tailoring literature [33], examining moderators of preferences may be an efficient way to develop tailored interventions.

Interestingly, some individuals preferred more negative messaging. As there is ample evidence that aggression or a demeaning tone decreases long-term adherence (as opposed to constructive negative feedback or consequence-oriented messaging, which can be useful), this finding highlights the downside of preference research in guiding intervention design. Communications that contain negative components like shaming or punishment are contraindicated in interventions to promote long-term behavior change [34], even if some people claim to prefer these messages. So why do some individuals prefer impolite or aggressive messages and how can we identify this group? For example, post-hoc analysis revealed that the small group who preferred the sad emoticon also preferred more aggressive messaging. Similarly, we found that individuals who endorsed being generally sad were significantly more likely than those who reported being happy to choose consequence-oriented and literal messages, and there was a trend for choosing “I” messages over “We” messages. In cases such as this, preferences might simply be an assessment tool to understand the individual, and tailoring to these types of preferences may not improve outcomes. At the same time, it is possible that sending messages congruent with preferences can increase engagement in an intervention. Then as the user becomes more engaged, there can be a slow and subtle shift to “healthier” messaging. For example, an intervention can send individuals with depression messages with “I” statements early in the change process and then slowly shift to “We” statements as the intervention progresses. The general tailoring literature further indicates that engagement in digital interventions is one of the best predictors of outcome [35].

It was surprising to us that there were few differences in preferences between those with different scores on process rulers, with the only differences being that those with higher confidence, importance, and benefits to changing preferred caps emphasis in messages to no emphasis and those who saw greater benefits of meeting their goal preferred coaching messages when compared to those who perceived few benefits in meeting their goal. Both messaging types are designed to illicit some sort of emotion in the individual and it could be that adding a positive emotional emphasis is useful for individuals who see greater benefits or importance for change or have greater confidence in their ability to change. While understanding these processes certainly has important implications for message development, it was more striking to us that there were so few differences and results should be interpreted with caution.

### Limitations

Despite the promise of this line of research, there are limitations. The most salient is that preferences do not necessarily translate into improved outcomes [36] and may, in fact, reflect the underlying negative schemas of an individual, such as a tendency to self-shame or diminish self-efficacy. Therefore, regardless of preferences, certain precautions should be taken when individualizing messages, such as avoiding aggressive language or sad emoticons, whereas more leeway can be given with messages that are neutral or moderately contraindicated (eg, fostering an external locus of control) if it improves engagement. Another limitation is that we did not send actual mobile messages, but asked for preferences in an online survey. It is possible that viewing a message on a computer screen as opposed to in an actual text or mobile alert may yield different preferences. This is particularly true of preferences for variables like *textese*, which are most commonly seen in mobile communications. Nevertheless, our results are congruent with much of the general health communications literature in terms of outcomes [37], indicating that preferences research is a useful preliminary step in intervention development. Results of our moderator analyses should be interpreted with reservation as we did multiple analyses, inflating the possibility of a Type-II error. However, we performed logistic regression with significant variables to look at the unique contributions of each moderator to messaging preferences.

There were population limitations as well. Namely, we did not restrict the availability of the survey only to people who might be the target of a health intervention, but left it open to a wider population with a broad range of goals—some of which were completely unrelated to health. It is possible that this wider population may have different message preferences than a sample of people who are struggling specifically with a health or mental health problem. Because some of the message groupings were added during later cohorts, there were smaller sample sizes for these preference findings, reducing the strength of the effects. Therefore, future studies should replicate these findings with larger samples. Finally, we limited this analysis to US populations. As a larger part of the study, we are comparing US and Indian populations on messaging preferences, as there is good evidence that linguistic styles and communications differ dramatically between cultures [38]. Despite some limitations, using bottom-up participatory strategies can help us design interventions that account for the client’s preferences within a larger theoretical framework. Adding quantitative preference testing yields similar results as focus groups with significantly lower burden, and can be iterated and modified quickly to test subtle variations in our models of care.

### Conclusions and Future Research

When taken together, understanding preferences for intervention presentation may improve engagement, regardless of the content or theory upon which an intervention is based [39-41], and is therefore a logical extension of mobile messaging research. While we do not know the impact of differentially structuring messages on actual outcomes, they may increase engagement in message-based interventions. For example, a recent study revealed that while emoticons did increase users’ enjoyment of the texting interaction, the perceived enjoyment of emoticons had no effect on the perceived usefulness of the message [42]. Once we understand these global messaging preferences, we can begin to further examine preference moderators such as those reported in the health tailoring literature [11,12]. Moreover, research should examine user preferences for actual text messages using ecological momentary assessment and response rate feedback. This research will provide investigators with a better understanding of preferences in real-world contexts. Future research can also focus on receptivity as it relates to specific periods in the change process (eg, an emoticon when one is meeting one’s goal compared to an emoticon when one is not). Muench and colleagues [23] revealed that individuals generate different messages for different critical points in the change process (eg, when someone is at risk for relapse vs someone who has already relapsed), underscoring that in dynamically tailored interventions, messages must contain appropriate content at appropriate moments, and this may apply to message structure as well. Using consumer research methods such as rapid iterative design, in which surveys are republished multiple times with slight changes to content, can expedite intervention development research. Because just-in-time mobile interventions are in their infancy, this line of research can help guide researchers to test proposed intervention components prior to conducting larger scale trials. As a preliminary step, we developed general guidelines for writing messages for goal-directed behavioral messaging interventions. We purposely kept it short and presented the most robust findings. However, this should be considered preliminary until more research can be done.

## Multimedia Appendix 1

Tips for writing intervention messages based on user preferences.

## Abbreviations

CHERRIES: Checklist for Reporting Results of Internet E-Surveys
HIT: human intelligence task
MTurk: Amazon Mechanical Turk
SMS: short message service
