# Supplementary material for: Understanding Messaging Preferences to Inform Development of Mobile Goal-Directed Behavioral Interventions
Source: J Med Internet Res. 2014 Feb 5;16(2):e14. doi: 10.2196/jmir.2945 (PMC3936297; doi:10.2196/jmir.2945)
Supplement: Supplementary file 1 [file jmir_v16i2e14_app1.pdf]

Tips for Writing Intervention Messages Based On User Preferences

1. Use proper grammar.
2. Avoid using “textese” (eg, LOL, thx).
3. Avoid including sad emoticons.
4. Discuss the benefits of changing as often as or more often than the consequences of not changing.
5. Shorter messages with one point are useful, but don’t sacrifice content for brevity.
6. Be polite and use *please* and *thank you* when possible.
7. Be directive without being aggressive. Avoid using passive language such as “You might want to try…”
8. Avoid being sarcastic or aggressive in messages.
9. If you intend to include humor in a message, remember that various types of humor are received differently by different people, and do not always translate well.
10. Punctuation like exclamation marks can enhance a friendly or encouraging tone in messages, but should be restricted to use in appropriate contexts.
11. Emoticons can enhance a friendly or encouraging tone in messages, but should be restricted to use in appropriate contexts and may be better received by certain groups.
12. Declarative statements are typically preferred to questions that require the user to evaluate their goals. However, evaluative statements may be helpful in some instances.
13. When citing statistics, it is better to cite a robust or powerful statistic. If results are positive but not extremely robust, a general statement without the statistic may be sufficient.
14. Highlight internal locus of control.
15. Messages that are from an individual (*I, me*) as opposed to a group (*we, us*), or are directed at an individual (*you*) rather and a group (*we, everyone*) are generally preferred. However, affirmations that include *we* statements to imply social support may be useful and may be more acceptable to individuals under 40 versus those over 40.
16. Capitalizing words or phrases to add emphasis is useful in some scenarios, but should be included in only a few messages with important content. Try not to capitalize a command or negative statement. If you capitalize, be positive and use it for coaching emphasis.
17. Metaphors and imagery can also be used to add meaning to a message, but keep in mind that certain individuals may not understand the metaphor or prefer more literal language.
18. Understand your target population and their goals and messaging preferences.

Note: These suggestions are based on the current preferences data referenced in this paper and do not represent conclusive findings and guidance for developing messages for goal-directed behaviors. Please consult additional resources, such as the CDC's *Guide to Writing for Social Media* [27].
